# *MYC* amplification in leukemia detected by cytogenetics

**DOI:** 10.1002/jha2.189

**Published:** 2021-04-08

**Authors:** Roberta Maria da Silva Oliveira Safranauskas, Maria Gabriella Cordeiro, Elvira Deolinda Rodrigues Pereira Velloso

**Affiliations:** ^1^ Hospital Israelita Albert Einstein São Paulo Brazil

**FIGURE 1:**
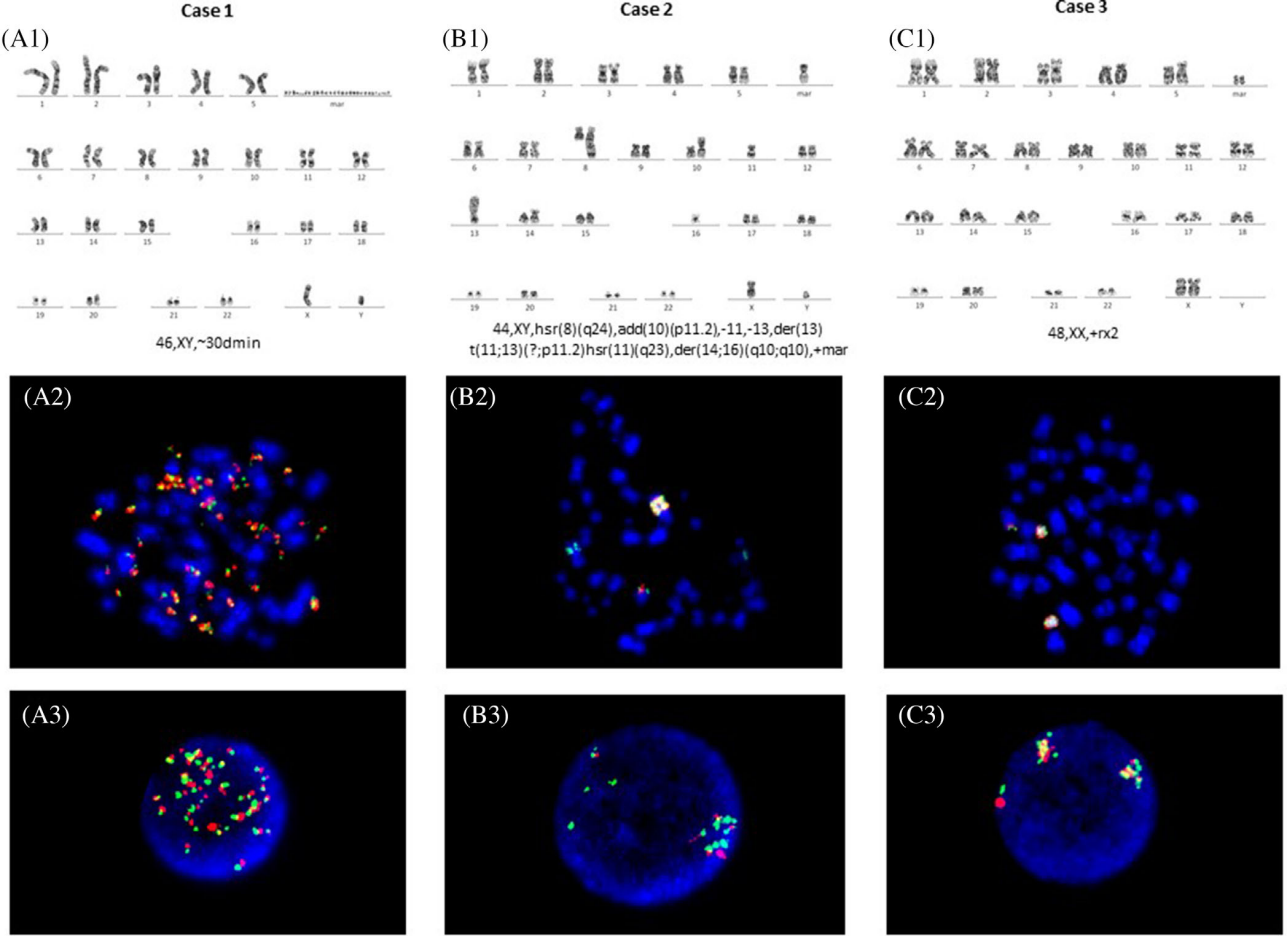


Cytogenetic studies of three patients aged between 65 and 76 years with newly de novo acute myeloid leukemia, AML, NOS according to WHO classification (one case AML without maturation, one case acute myelomonocytic leukemia and one case AML with maturation). G‐band karyotyping shows the presence of double minutes (dmin, Panel A1), homogeneously staining regions (hsr, Panel B1), and ring chromosome (Panel C1) (Figure 1). Fluorescence in situ hybridization using *MYC* probe Dual Color, Break apart shows *MYC* amplification in all cases (metaphasic FISH panels A2, B2, C2 and interphasic FISH panels A3, B3, C3) (Figure 1).

Extrachromosomal dmin and intrachromosomal hsr are hallmarks of genetic amplification in cancer. Ring chromosomes are also vehicles of genes amplification.

Although they are often found in solid neoplasm, they are rare in leukemia, been detected in less than 1% of AML with abnormal karyotype, and mostly associated with AML with myelodysplastic‐related changes and therapy‐related AML, older age, complex karyotype, and poor prognosis. They could also be detected in myelodysplastic syndromes, chronic myelomonocytic leukemia, and chronic myeloid leukemia in blast‐phase at diagnosis or during the course of the disease.

In hematological neoplasm, these cytogenetic elements carry *MYC* (8q24.21) *or MLL (KMT2A–*11q23) amplification. Although the mechanisms underlying their genesis are poorly understood, the excision of a DNA segment followed by its circularization and “head‐to‐tail” amplification (episome model) is the most acceptable. The amplified *MYC* is upregulated and could be detected by immunohistochemical staining on bone marrow core biopsies.

